# A phase II, multicentre trial of decitabine in higher-risk chronic myelomonocytic leukemia

**DOI:** 10.1038/leu.2017.186

**Published:** 2017-07-07

**Authors:** V Santini, B Allione, G Zini, D Gioia, M Lunghi, A Poloni, D Cilloni, A Sanna, E Masiera, M Ceccarelli, O Abdel-Wahab, A Terenzi, E Angelucci, C Finelli, F Onida, A Pelizzari, D Ferrero, G Saglio, M Figueroa, A Levis

**Affiliations:** 1 Department of Hematology, AOU Careggi, University of Florence, Florence, Italy; 2 AOU Citta della Salute e della Scienza, Torino, Italy; 3 Department of Oncology and Hematology, Hematology Institute, Fondazion e Policlinico Gemelli, UCSC, Rome, Italy; 4 Division of Haematology, Department of Translational Medicine, UPO, Novara, Italy; 5 Department of Hematology, AOU Ospedali Riuniti, Università Politecnica Marche, Ancona, Italy; 6 Department of Clinical and Biological Sciences, San Luigi Hospital, University of Turin, Turin, Italy; 7 Università degli studi di Firenze, Dipartimento di medicina sperimentale e Clinica, Firenze, Italy; 8 AOU Città della salute e della scienza di Torino, Torino, Italy; 9 Human Oncology and Pathogenesis Program, and Leukemia Service, Department of Medicine, Memorial Sloan Kettering Cancer Center, New York, NY, USA; 10 BMT Centre of Perugia, Department of Hematology, Perugia, Italy; 11 Hematology and Transplant Unit, Ospedale Oncologico di Riferimento Regionale Armando Businco, Cagliari, Italy; 12 Institute of Hematology, S.Orsola-Malpighi University Hospital, Bologna, Italy; 13 Oncohematology Unit, Fondazione IRCCS Ca Granda Ospedale Maggiore Policlinico—Department of Oncology and Hemato-Oncology, University of Milan, Milan, Italy; 14 Spedali Civili Brescia Hematology Unit, Brescia, Italy; 15 Hematology Division, Università degli Studi di Torino, Torino, Italy; 16 Department of Clinical and Biological Sciences, University of Turin, Torino, Italy; 17 Department of Human Genetics and, Sylvester Comprehensive Cancer Center, University of Miami Miller School of Medicine, Miami, FL, USA

## Abstract

Chronic myelomonocytic leukemia (CMML) is a complex clonal hematological disorder classified among myelodysplastic (MDS)/myeloproliferative neoplasms. Prognosis is poor and there is a lack of effective treatments. The hypomethylating agent decitabine has shown activity against MDS and elderly acute myeloid leukemia, but there is little data focusing specifically on its efficacy in CMML. In this prospective, phase 2 Italian study, CMML patients received intravenous decitabine 20 mg/m^2^ per day on Days 1–5 of a 28-day treatment cycle. Response was evaluated after four and six cycles; patients responding at the end of six cycles could continue treatment with decitabine. Forty-three patients were enrolled; >50% were high-risk according to four CMML-specific scoring systems. In the intent-to-treat population (*n*=42), the overall response rate after six cycles was 47.6%, with seven complete responses (16.6%), eight marrow responses (19%), one partial response (2.4%) and four hematological improvements (9.5%). After a median follow-up of 51.5 months (range: 44.4–57.2), median overall survival was 17 months, with responders having a significantly longer survival than non-responders (*P*=0.02). Grade 3/4 anemia, neutropenia and thrombocytopenia occurred in 28.6%, 50% and 38% of patients, respectively. Decitabine appears to be an effective and well-tolerated treatment for patients with high-risk CMML.

## Introduction

Chronic myelomonocytic leukemia (CMML) is a complex clonal hematological disorder that is classified by the World Health Organization among myelodysplastic (MDS)/myeloproliferative neoplasms.^1^ The 2016 revision to the World Health Organization classification of tumors of the hematopoietic and lymphoid tissues describes three categories of CMML based on blast count:^2, 3^ CMML-0 (<2% peripheral blasts and <5% bone marrow blasts), CMML-1 (2–4% peripheral blasts and/or 5–9% bone marrow blasts) and CMML-2 (5–19% peripheral blasts, 10–19% bone marrow blasts and/or presence of Auer rods). Before this revision, patients with <2% peripheral blasts and <5% bone marrow blasts were included in the CMML-1 category.^4^

Diagnosis can be difficult, requiring a combination of morphologic, histopathologic and cytogenetic approaches.^5^ The World Health Organization diagnostic criteria for CMML are as follows:^2, 3^ persistent monocytosis⩾1 × 10^9^/l; no Philadelphia chromosome or *BCR-ABL1* fusion gene; exclusion of primary myelofibrosis, polycythemia vera and essential thrombocytothemia; no *PDGFRA*, *PDGFRB* or *FGFR1* rearrangements, or *PCM1-JAK2* fusions if eosinophilia present; <20% blasts in peripheral blood and bone marrow; and dysplasia in one or more myeloid lineages. If myelodysplasia is absent or minimal, a diagnosis of CMML can still be made if a cytogenetic abnormality is present in the hematopoietic stem cell, or if monocytosis has persisted for more than 3 months with all other possible causes excluded.

Significant heterogeneity makes prognosis in CMML difficult to estimate, but in general it is poor. Commonly used for MDS, the original and revised International Prognostic Scoring Systems^6, 7^ are not suitable for CMML, because they exclude patients with proliferative disease. Newer prognostic models (such as the CMML-specific prognostic scoring system,^8, 9^ Groupe Francophone de Myelodysplasies (GFM) model^10^ and the Mayo Molecular Model^11^) take cytogenetics and somatic mutations into account. Very recently, an integrated prognostic scoring system has been proposed that takes clinical parameters, cytogenetics and somatic mutations into account.^12^

The only potentially curative treatment option for CMML is hematopoietic stem cell transplant, but this is not suitable for many patients because of their age and comorbidities. There are currently no prospective data on the benefits and risks of hematopoietic stem cell transplant in CMML. Management usually focuses on supportive care and cytoreductive therapy, depending on whether the disease is dysplastic or myeloproliferative.^13^ Hydroxyurea is currently a mainstay therapy for proliferative disease.^14^

The hypomethylating agents (HMAs) azacitidine and decitabine have been shown to be active in MDS patients in randomized phase 3 trials.^15, 16, 17^ However, the numbers of CMML patients in these trials were limited and their results were not reported separately. In two retrospective analyses of decitabine, overall response rates (ORRs) ranged from 26 to 68% and 2-year survival from 25 to 48%.^18, 19^ In a prospective phase 2 study in which 39 CMML patients received 20 mg/m^2^ decitabine per day on days 1–5 of 28-day cycles, the ORR was 38% and 2-year survival was 48%.^20^

The European Medicines Agency has approved azacitidine for the treatment of non-proliferative CMML (white blood cell (WBC) count <12 000), but HMAs are not currently a licensed option for treating proliferative forms. In Italy, several national societies recommend that patients with myelodysplastic-type CMML and ⩾10% bone marrow blasts should be managed with supportive therapy in combination with HMAs.^13^ Alongside the lack of specific treatment options, CMML-specific response criteria were not used in any clinical trials, having only been recently developed by Savona *et al.*^21^

Here we report the results of a prospective phase 2 study that assessed the efficacy and safety of decitabine in Italian CMML patients.

## Methods

### Study design and patients

This was an open-label, phase 2 study carried out at 15 centres across Italy between April 2010 and October 2011. Patients aged ⩾18 years with a diagnosis of CMML according to World Health Organization criteria,^4^ an Eastern Cooperative Oncology Group performance status ⩾2 and a life expectancy ⩾6 months were eligible to enter the study. CMML patients were classified according to FAB^22^ as dysplastic CMML (MDS-CMML) when WBC counts ⩽13 000/mm^3^ or proliferative (myeloproliferative neoplasm-CMML) when WBC >13 000/mm^3^. Patients with a WBC count ⩽12 000/mm^3^ were required to have International Prognostic Scoring System intermediate-2 risk. Those with a WBC count >12 000/mm^3^ had to have at least two of the following: blast cells >5% in bone marrow, a cytogenetic abnormality other than t(5;12) (q33;p13), anemia (that is, Hb <10 g/dl), thrombocytopenia (that is, platelets <100 000/mm^3^), splenomegaly (>5 cm below the costal margin) and extramedullary localization. Patients with a myeloproliferative or myelodysplastic syndrome other than CMML and those who had acute blastic transformation of CMML with bone marrow blast cells >20% were excluded. Other exclusion criteria included eligibility for allogenic stem cell transplant with an identified donor, CMML with t(5;12) or *PDGFBR* rearrangement, intensive chemotherapy in the last 3 months and previous treatment with a HMA. Patients were eligible if untreated or previously treated with hydroxyurea or etoposide given orally, or non-intensive chemotherapy or intensive chemotherapy given more than 3 months before inclusion. Patients received intravenous decitabine (Dacogen; Janssen Pharmaceutica NV, Beerse, Belgium) 20 mg/m^2^ per day on Days 1–5 of a 28-day treatment cycle. Discontinuation was allowed at the patient’s request or if they experienced progression with blastic transformation, grade 3/4 toxicity according to National Cancer Institute criteria (except cytopenia) or other changes in their condition that the investigator felt warranted removal of the patient from the study.

After a minimum of four treatment cycles, patients were assessed for response to treatment. Responders were defined as patients who achieved hematological improvement or better according to International Working Group 2006 criteria;^23^ these patients continued treatment for a further two cycles. Minor responders and patients with stable disease were allowed to continue in the study at the investigator’s discretion. Patients with progressive disease were discontinued from the study. Patients who completed all six treatment cycles were eligible to receive maintenance treatment with decitabine. After completion of, or discontinuation from, the study, patients were followed up every 4 months.

The study was carried out in accordance with the Declaration of Helsinki. All patients provided written informed consent and all participating trial sites gained approval from the relevant local ethics committee. This study is registered on ClinicalTrials.gov (NCT01251627).

### Objectives and outcome measures

The primary aim of the study was to assess the efficacy of decitabine in the treatment of CMML. The primary outcome measure was the ORR, defined as the proportion of patients achieving a complete response, marrow complete response, partial response or hematological improvement. Secondary outcome measures included overall survival (OS), event-free survival, duration of response, the number of blood and platelet transfusions, the number of days of hospitalization and safety.

### Somatic mutations

Bone marrow samples were collected before treatment and DNA was extracted from unsorted mononuclear cells. The methods used for mutational sequencing have been published previously.^24^ Briefly, target regions (exons plus splice junctions) were captured and 500 ng of DNA from each sample was quantified and sequenced using paired-end sequencing. Sequences were aligned to the human genome and the Genome Analysis Toolkit^25^ was used to perform further local indel alignment and base-quality score recalibration, and to generate single-nucleotide variation and indel calls. Variants with functional consequence on genes were annotated and their presence identified in dbSNP 137, the 1000 Genomes Project, ESP6500 (the National Heart, Lung and Blood Institute GO Exome Sequencing Project) and COSMIC 67.

### Statistical analyses

Conventional treatments for CMML (hydroxyurea or etoposide) give ORRs of no more than 15%. This study was designed to detect a clinically relevant 20% increase in ORR with decitabine (that is, from 15 to 35%) with 85% power and a significance level of 0.05. The planned sample size for this single-stage Fleming-A’Hern phase 2 design was 39 patients. Achievement of OR by ⩾11 patients after six cycles was to be considered sufficient to justify further investigation. To take into account losses to follow-up for time-to-event endpoints, the sample size was increased by 10 and 43 patients were enrolled.

Primary efficacy and safety data were analyzed for the intent-to-treat population, that is, all patients who received at least one dose of decitabine. Discrete variables were summarized by frequency and percentage. Continuous variables were summarized by mean and s.d. or median and interquartile range.

OS was defined as the time from enrolment to death from any cause or last follow-up evaluation. Event-free survival was defined as the time from enrolment to progression, transformation to acute myeloid leukemia or death from any cause. Duration of response was defined as the time from clinical response to progression, transformation to acute myeloid leukemia or death from any cause. These time-to-event endpoints were analyzed using the Kaplan–Meier method.

Adverse events (AEs) were reported by type and grade according to the Common Terminology Criteria for Adverse Events (version 3.0).

## Results

### Patients

Between April 2010 and October 2011, 43 patients were enrolled at 15 sites across Italy. The intent-to-treat population included 42 patients; their baseline characteristics are shown in Table 1. Most patients were male (71.4%) and two-thirds had proliferative CMML. Between 76 and 93% of patients were high- or intermediate-risk (depending on the prognostic scoring system applied retrospectively, as developed after inception of this study; Table 1) and 15/42 had an *ASXL1* mutation.

**Figure 1:**
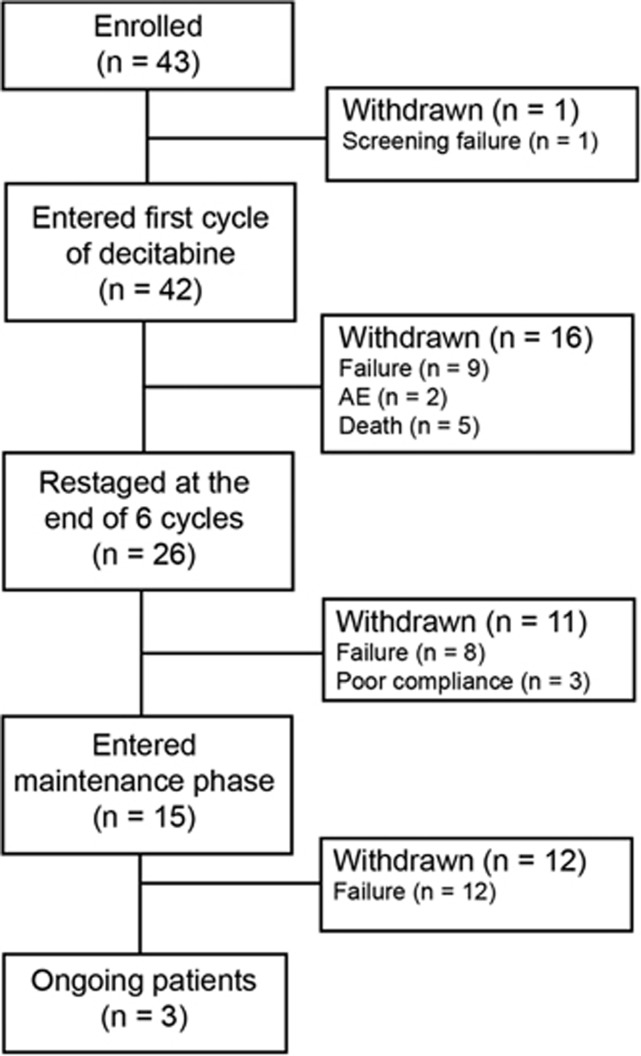
Patient disposition. Flow of patients from enrolment to time of analysis.

Figure 1 shows the flow of patients through the study. The median number of treatment cycles was 6 (range: 1–34). Twenty-six patients (62%) received all six cycles; the most common reasons for discontinuation were treatment failure (*n*=9) and death (*n*=5).

### Somatic mutations

The results of the analysis of the most frequent somatic mutation found in CMML were not possible in all cases in this study and have already been published previously in a study in which a subset of the CMML patients treated with decitabine was analysed for methylation pattern, gene expression profile and the presence of somatic mutations.^24^ The incidence and type of mutation at diagnosis is presented in Table 1. The most frequent mutations as expected were those of SRSF2 (45.2%), TET2 (38.1%) and ASXL1 (35.7%).

There was no correlation between the presence of a single mutation and pattern of response to decitabine.

### Response rates

In the intent-to-treat population, the ORR was achieved in 20 patients (47.6% lower 90% confidence interval (CI): 34.2%) (Table 2). Patients with CMML-1 had a higher ORR than those with CMML-2 (53.8% vs 37.5%, *P*=0.09). The ORR was also higher in patients with dysplastic CMML than in those with proliferative CMML (64.3% vs 39.3%, *P*=0.12). Regarding reduction of organomegaly, only 5/20 of responsive patients experienced a decrease in spleen size.

### Survival and progression

**Figure 2:**
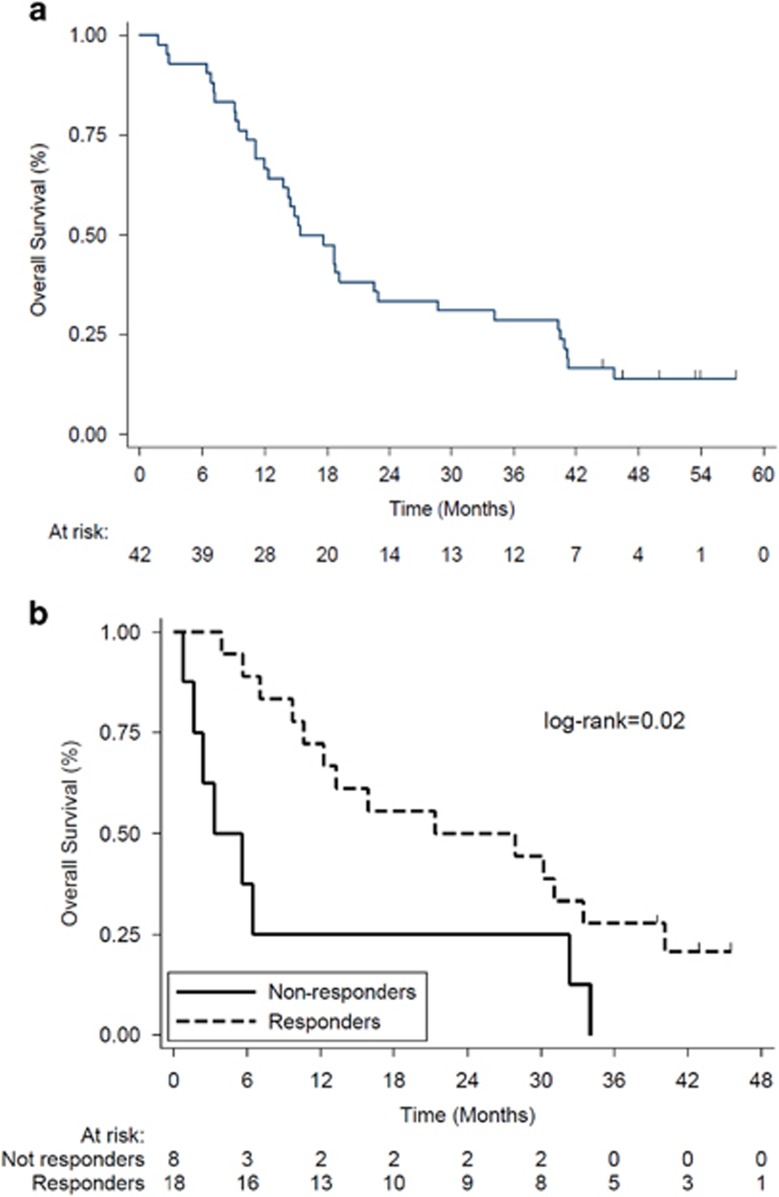
Overall survival. Kaplan–Meier curves showing OS in (**a**) the intent-to-treat (ITT) population and (**b**) responders vs non-responders. Vertical lines denote censored patients.

**Figure 3:**
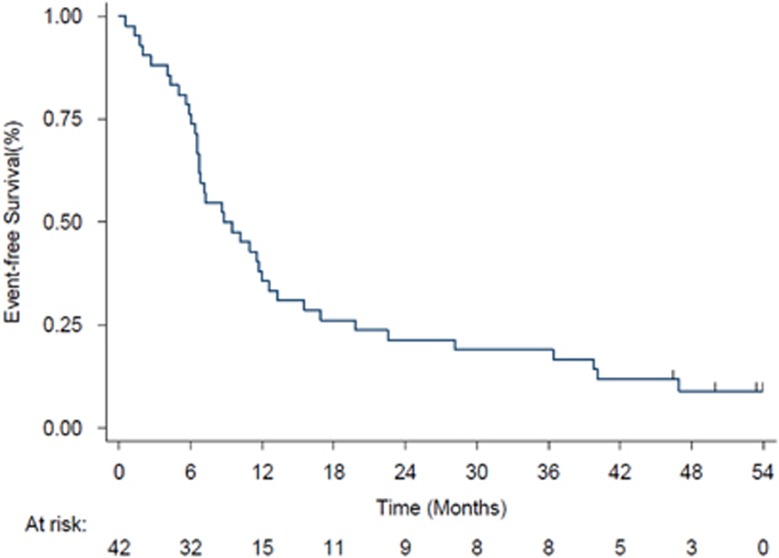
Event-free survival. Kaplan–Meier curves showing event-free survival in the intent-to-treat (ITT) population. Vertical lines denote censored patients.

**Figure 4:**
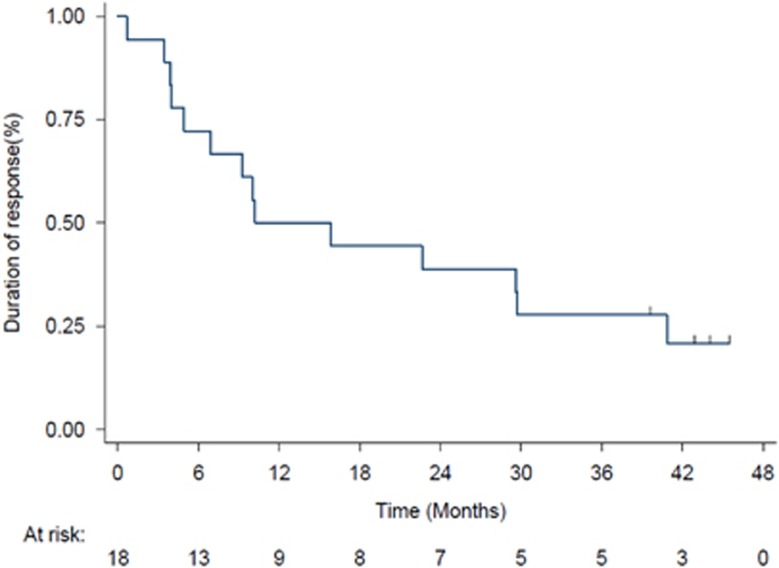
Duration of response. Kaplan–Meier curves showing duration of response. Vertical lines denote censored patients.

The median duration of follow-up was 51.5 months (range: 44.4–57.2). Median OS was 17 months (Figure 2a). The 1-year, 2-year and 3-year OS rates were 66.7% (95% CI: 50.3–78.7), 33.3% (95% CI: 19.8–47.5) and 28.6% (95% CI: 16.0–42.5), respectively. Patients who responded to treatment at the end of treatment had a significantly longer OS than those who did not (log-rank=0.02; Figure 2b). Specifically, median OS (months) was significantly different (*P*=0.0028): complete response: 31.08; marrow complete response: 10.59; hematological improvement: 21.3; partial response: not evaluable; stable disease: 2.36; progressive disease: 3.28. Median event-free survival was 8 months (Figure 3). The most common event was death, which occurred in 36 patients (85.7%). Thirty-two patients (76.2%) progressed and 24 (57.1%) had transformation to acute myeloid leukemia. The 1-year, 2-year and 3-year event-free survival rates were 35.7% (95% CI: 21.7–49.9), 21.4% (95% CI: 10.6–34.7) and 19.1% (95% CI: 8.9–32.0), respectively. The median duration of response after six cycles was 10 months (Figure 4). At 1-year, 52.6% (95% CI: 28.7–71.9) of responders were still responding to treatment; the corresponding figures at 2 and 3 years were 42.1% (95% CI: 20.4–62.5) and 26.3% (95% CI: 9.6–46.8), respectively. In this group of patients, 15/42 carried ASXL1 mutations. OS for mutated patients was 17.6 months vs 14.4 months for patients without ASXL1 mutation (*P*=0.76). There was a significant difference in OS (*P*<0.001) according to baseline methylation pattern: patients who had predictive signature^24^ showed 23.03 months median OS vs 11.2 months median OS of patients without predictive signature. Median OS after decitabine treatment discontinuation was 3.28 months. Two patients underwent hematopoietic stem cell transplant, one ASXL1-mutated responder (transplanted at relapse) and one ASXL1 unmutated, resistant to decitabine treatment. Their OS was 45.63 and 44.67 months, respectively. These patients were not censored in the global OS evaluation.

### Transfusions and hospitalizations

At baseline, 18 patients (42.9%) required transfusions. During the treatment period, transfusions were carried out during 117/210 cycles; 39 patients needed at least one transfusion. Nine out of 18 patients became transfusion independent. During follow-up, 21 patients needed at least one transfusion. During the treatment period, 9 patients had a total of 24 scheduled hospital admissions. The median length of hospitalization was 6.5 (range 1.0–31.0) days. Ten patients had a total 14 unscheduled hospital admissions: 4 caused by infective disease, 3 stroke, 3 trauma, 1 thrombocytopenia, 1 heart failure, 1 suspected pulmonary embolism and 1 disease progression. None of the hospitalizations was related to the drug according to the treating physicians. During follow-up, seven patients each had one unscheduled hospital admission.

### Safety

The most common AEs during treatment were hematological: thrombocytopenia, anemia and neutropenia (Table 3). In 50% of cases, anemia was grade 3 or 4. More than three-quarters of thrombocytopenia cases and over 80% of neutropenia cases were grade 3 or 4. The most common non-hematological AEs were gastrointestinal; all of these were grade 1 or 2. Two patients had grade 5 AEs: one cardiac event and one bleeding event. Thirty-six patients died: 5 during the 6-month study period and 31 during follow-up. In 29 of these patients, MDS was the cause of death.

## Discussion

CMML is a disease that is difficult to diagnose and has a poor prognosis. There are currently no effective treatments for patients who are unsuitable for hematopoietic stem cell transplant. Dysplastic and proliferative forms of CMML are likely to require different treatment approaches. Current recommendations are to treat dysplastic CMML with supportive care plus azacitidine and proliferative CMML with cytoreductive therapy to control proliferation and reduce organomegaly.^13^

In our study, decitabine induced a response in approximately half of patients, with responders having a survival advantage over non-responders. Although patients with CMML-2 and those with proliferative disease had lower response rates than those with CMML-1 or dysplastic disease, the results in these subgroups were encouraging. Decitabine was well tolerated in our elderly cohort; the incidence and type of AEs were as expected.

The ORR in our study is slightly higher than that of 38% reported in a previous study in 39 CMML patients conducted by the GFM.^20^ Whereas we found CMML-1 patients to be more likely to respond to treatment, the GFM CMML study showed the opposite, with 50% of CMML-2 patients responding, compared with 17.6% of CMML-1 patients. Median OS was similar in our study and the GFM CMML study, but 2-year OS was lower in our study (33.3% vs 48%).

Previous studies in mixed cohorts of patients with MDS and related malignancies have linked increased response to HMAs to mutations in *TET2*^26, 27^ (particularly when *ASXL1* is not mutated^27^) and *DNTM3A*.^26^ However, in a cohort of 40 patients from the present study, we found that no somatic mutation, including *ASXL1*, was predictive of response to decitabine in CMML.^24^ Likewise, the GFM CMML study also found no association between response to decitabine and mutational status.^20^ This may indicate a difference in the impact of mutational status between patients with CMML and those with other myeloid malignancies. In addition, the studies showing an association between somatic mutations and response included patients who received azacitidine, as well as patients who received decitabine.^26, 27^

Although somatic mutations did not differentiate responders from non-responders in our cohort, we found a pattern of 167 differentially methylated regions of DNA that was predictive of response.^24^ Using this, we developed an epigenetic classifier that can accurately predict response to decitabine at the time of diagnosis. It can take several cycles of treatment before it becomes apparent whether the patient will respond or not; this classifier would allow potential non-responders to be identified early and put onto an alternative treatment, rather than having to endure months of fruitless treatment with decitabine.

Many of the patients in our cohort were high-risk according to the prognostic scoring systems used; thus, we could not determine whether high- and low-risk patients have a differential sensitivity to decitabine. The median OS was 17 months, which compares favorably with best supportive care and hydroxyurea.^9, 10^ Such *et al.* used a cohort of patients receiving best supportive care when developing the CMML-specific prognostic scoring system.^9^ Patients who fell into the high-risk category had a median OS of 5–9 months.^9^ Patients classed as high-risk according to the GFM prognostic scoring system had a median OS of 14.4 months.^10^ Most patients in this latter study were receiving best supportive care, but hydroxyurea and HMAs were also used. High-risk patients according to the Mayo Molecular Model had a median OS of 16 months; the authors do not report what treatment(s) the patients were receiving.^11^ Evaluation on whether the stratification in single categories of risk according to the specific models resulted in difference in response was not possible because of the small numbers. According to CMML-specific prognostic scoring system and GFM scores, the differences in survival among groups was maintained after decitabine, whereas according to Mayo scores there were no differences in OS after treatment.

Decitabine appears to be an effective treatment for patients with high-risk CMML, including those with proliferative disease. Further research is needed to determine whether there is a difference in response between low- and high-risk patients. Owing to the rarity of CMML, large, specific trials can be difficult to conduct. However, we are currently conducting (within the guidance of the European MDS Studies Coordination Office) an international, randomized, phase 3 trial comparing decitabine (±hydroxyurea) with hydroxyurea in patients with advanced proliferative CMML (ClinicalTrials.gov identifier: NCT 02214407). The results of this trial will provide further important insights into the efficacy of decitabine as a treatment for CMML, particularly in patients with proliferative disease, for whom treatment with HMAs is currently not a licensed option.

## Figures and Tables

**Table 1:** Baseline characteristics (ITT population)

Patients, *n*	42
Median age, years (range)	71.5 (42–84)
Male/female, *n* (%)	30/12 (71.4/28.6)
CMML-1/CMML-2^a^, *n* (%)	26/16 (61.9/38.1)
dCMML/pCMML, *n* (%)	14/28 (33.3/66.7)
Hb g/dl, median (IQR)	9.8 (9.1–11.0)
AMC × 10^9^/l, median (IQR)	3.39 (2.23–7.25)
WBC × 10^9^/l, median (IQR)	18.6 (13.9–28.1)
PLT × 10^9^/l, median (IQR)	54.5 (34.0–75.0)
Bone marrow blasts %, median (IQR)	6.0 (3–12)
	
*Cytogenetics,* n *(%)*	
Altered	12 (28.6)
Normal	28 (66.7)
Not evaluable	2 (4.7)
Splenomegaly, *n* (%)	22 (52.4)
Hepatomegaly, *n* (%)	19 (45.2)
Lymphadenomegaly, *n* (%)	6 (14.3)
Marrow fibrosis, *n* (%)	13 (30.9)
	
*ASXL1,* n *(%)*	
Mutated	15 (35.7)
Not evaluable	4 (9.5)
	
*SRSF2,* n *(%)*	
Mutated	19 (45.2)
Not evaluable	5 (11.9)
	
*TET2,* n *(%)*	
Mutated	16 (38.1)
Not evaluable	5 (11.9)
	
*P53,* n *(%)*	
Mutated	3 (7.1)
Not evaluable	5 (11.9)
	
*MMM prognostic risk categories,* n *(%)*	
High risk	13 (30.9)
Int-2	14 (33.3)
Int-1	10 (23.8)
Not evaluable	5 (12.0)
	
*CPSS prognostic risk categories,* n *(%)*	
High risk	3 (7.1)
Int-2	20 (47.6)
Int-1	15 (35.7)
Low	2 (4.8)
Not evaluable	2 (4.8)
	
*Mayo prognostic risk categories,* n *(%)*	
High risk	26 (61.9)
Int	13 (30.6)
Low	3 (7.1)
	
*GFM prognostic risk categories,* n *(%)*	
High	14 (33.3)
Int	18 (42.9)
Low	10 (23.8)

Abbreviations: AMC, absolute monocyte count; CMML, chronic myelomonocytic leukemia; dCMML, dysplastic CMML; CPSS, CMML-specific prognostic scoring system; GFM, Groupe Francophone de Myelodysplasies; Hb, hemoglobin; IQR, interquartile range; ITT, intent-to-treat; MMM, Mayo Molecular Model; PLT, platelet; pCMML, proliferative CMML; WBC, white blood cells; WHO, World Health Organization.

Percentages may not total 100 owing to rounding.

a Defined according to the 2008 edition of the WHO classification of tumors of the hematopoietic and lymphoid tissues.

**Table 2:** Overall clinical response (end of cycle 6 or at early withdrawal)

	*Number (%) of patients*				
	*ITT (*n=*42)*	*CMML-1*^a^ (n=*26)*	*CMML-2*^a^ (n=*16)*	*dCMML (*n=*14)*	*pCMML (*n=*28)*
ORR	20 (47.6)	15 (57.6)	5 (31.25)	9 (64.3)	11 (39.3)
CR	7 (16.6)	5 (19.2)	2 (12.5)	3 (21.4)	4 (14.3)
mCR	8 (19.0)	6 (23.1)	2 (12.5)	4 (28.6)	4 (14.3)
PR	1 (2.4)	0 (0.0)	1 (6.2)	0 (0.0)	1 (3.5)
HI	4 (9.5)	4 (15.3)	0 (0.0)	2 (14.2)	2 (7.2)
SD	9 (21.4)	4 (15.3)	5 (31.3)	0 (0.0)	9 (32.1)
PD	13 (31.0)	7 (26.9)	6 (37.5)	5 (35.7)	8 (28.6)

Abbreviations: CR, complete response; CMML, CMML, chronic myelomonocytic leukemia; dCMML, dysplastic CMML; HI, hematological improvement; ITT, intent-to-treat; ORR, overall response rate; mCR, marrow CR; pCMML, proliferative CMML; PD, progressive disease; PR, partial remission; SD, stable disease; WHO, World Health Organization.

Percentages may not total 100 owing to rounding.

a Defined according to the 2008 edition of the WHO classification of tumors of the hematopoietic and lymphoid tissue.

**Table 3:** Adverse events (ITT population; *n*=42)

	*Number (%) of patients*		
	*Any grade*	*Grade 3*	*Grade 4–5*
Anemia	24 (57.1)	11 (26.2)	1 (2.4)
Thrombocytopenia	27 (64.3)	4 (9.5)	17 (63.1)
Neutropenia	19 (45.2)	7 (18.7)	9 (21.4)
Cardiac	2 (4.8)	—	1^a^ (2.4)
Neurological	1 (2.4)	—	—
Gastrointestinal	10 (23.8)	—	—
Hepatic	2 (4.8)	1 (2.4)	—
Documented infection	6 (14.3)	2 (4.8)	1 (2.4)
Bleeding	9 (21.4)	—	1^a^ (2.4)
Febrile neutropenia	2 (4.8)	2 (4.8)	—
Other	8 (19.0)	1 (2.4)	—

Abbreviation: ITT, intent-to-treat.

a Grade 5 event.

## References

[bib1] Jaffe ES, Harris NL, Stein H, Vardiman JW (eds) World Health Organization Classification of Tumours. Pathology and Genetics of Tumours of Haematopoietic and Lymphoid Tissues. IARC: Lyon, France, 2001.

[bib2] Swerdlow SH et al (eds) World Health Organization Classification of Tumours of Haematopoietic and Lymphoid Tissues. IARC: Lyon, France, 2016; in press.

[bib3] Arber DA, Orazi A, Hasserjian R, Thiele J, Borowitz MJ, Le Beau MM et al. The 2016 revision to the World Health Organization classification of myeloid neoplasms and acute leukemia. Blood 2016; 127: 2391–2405. 27069254 10.1182/blood-2016-03-643544

[bib4] Swerdlow SH, Campo E, Harris NL, Jaffe ES, Pileri SA, Stein H et al (eds) World Health Organization Classification of Tumours of Haematopoietic and Lymphoid Tissues. IARC: Lyon, France, 2008.

[bib5] Parikh S, Tefferi A. Chronic myelomonocytic leukemia: 2013 update on diagnosis, risk stratification, and management. Am J Hematol 2013; 88: 968–974. 10.1002/ajh.23574 23963888

[bib6] Greenberg P, Cox C, LeBeau MM, Fenaux P, Morel P, Sanz G et al. International scoring system for evaluating prognosis in myelodysplastic syndromes. Blood 1997; 89: 2079–2088. 9058730

[bib7] Greenberg PL, Tuechler H, Schanz J, Sanz G, Garcia-Manero G, Solé F et al. Revised international prognostic scoring system for myelodysplastic syndromes. Blood 2012; 120: 2454–2465. 22740453 10.1182/blood-2012-03-420489 PMC4425443

[bib8] Such E, Cervera J, Costa D, Solé F, Vallespí T, Luño E et al. Cytogenetic risk stratification in chronic myelomonocytic leukemia. Haematologica 2011; 96: 375–383. 21109693 10.3324/haematol.2010.030957 PMC3046268

[bib9] Such E, Germing U, Malcovati L, Cervera J, Kuendgen A, Della Porta MG et al. Development and validation of a prognostic scoring system for patients with chronic myelomonocytic leukemia. Blood 2013; 121: 3005–3015. 23372164 10.1182/blood-2012-08-452938

[bib10] Itzkyson R, Kosmider O, Renneville A, Gelsi-Boyer V, Meggendorfer M, Morabito M et al. Prognostic score including gene mutations in chronic myelomonocytic leukemia. J Clin Oncol 2013; 31: 2428–2436. 23690417 10.1200/JCO.2012.47.3314

[bib11] Patnaik MM, Itzkyson R, Lasho TL, Kosmider O, Finke CM, Hanson CA et al. ASXL1 and SETBP1 mutations and their prognostic contribution in chronic myelomonocytic leukemia: a two-center study of 466 patients. Leukemia 2014; 28: 2206–2212. 24695057 10.1038/leu.2014.125

[bib12] Elena C, Gallì A, Such E, Meggendorfer M, Germing U, Rizzo E et al. Integrating clinical features and genetic lesions in the risk assessment of patients with chronic myelomonocytic leukemia. Blood 2016; 128: 1408–1417. 27385790 10.1182/blood-2016-05-714030 PMC5036538

[bib13] Onida F, Barosi G, Leone G, Malcovati L, Morra E, Santini V et al. Management recommendations for chronic myelomonocytic leukemia: consensus statement from the SIE, SIES, GITMO groups. Haematologica 2013; 98: 1344–1352. 24006407 10.3324/haematol.2013.084020 PMC3762089

[bib14] Patnaik MM, Tefferi A. Chronic myelomonocytic leukemia: focus on clinical practice. Mayo Clin Proc 2016; 91: 259–272. 26848006 10.1016/j.mayocp.2015.11.011

[bib15] Silverman LR, Demakos EP, Peterson BL, Kornblith AB, Holland JC, Odchimar-Reissig R et al. Randomized controlled trial of azacitidine in patients with the myelodysplastic syndrome: a study of the cancer and leukemia group B. J Clin Oncol 2002; 20: 2429–2440. 12011120 10.1200/JCO.2002.04.117

[bib16] Fenaux P, Mufti G, Hellstrom-Lindberg E, Santini V, Finelli C, Giagounidis A et al. Efficacy of azacitidine compared with that of conventional care regimens in the treatment of higher-risk myelodysplastic syndromes: a randomised, open-label, phase III study. Lancet Oncol 2009; 10: 223–232. 19230772 10.1016/S1470-2045(09)70003-8 PMC4086808

[bib17] Kantarjian H, Issa J-PJ, Rosenfeld CS, Bennett JM, Albitar M, DiPersio J et al. Decitabine improves patient outcomes in myelodysplastic syndromes. Results of a phase III randomized study. Cancer 2006; 106: 1794–1803. 16532500 10.1002/cncr.21792

[bib18] Aribi A, Borthakur G, Ravandi F, Shan J, Davisson J, Cortes J et al. Activity of decitabine, a hypomethylating agent, in chronic myelomonocytic leukemia. Cancer 2007; 109: 713–717. 17219444 10.1002/cncr.22457

[bib19] Wijermans PW, Rüter B, Baer MR, Slack JL, Saba HI, Lübbert M. Efficacy of decitabine in the treatment of patients with chronic myelomonocytic leukemia (CMML). Leuk Res 2008; 32: 587–591. 17881052 10.1016/j.leukres.2007.08.004

[bib20] Braun T, Itzkyson R, Renneville A, de Renzis B, Dreyfus F, Laribi K et al. Molecular predictors of response to decitabine in advanced chronic myelomonocytic leukemia: a phase 2 trial. Blood 2011; 118: 3824–3831. 21828134 10.1182/blood-2011-05-352039

[bib21] Savona MR, Malcovati L, Komrokji R, Tiu RV, Mughal TI, Orazi A et al. An international consortium proposal of uniform response criteria for myelodydplastic/myeloproliferative neoplasms (MDS/MPN) in adults. Blood 2015; 125: 1857–1865. 25624319 10.1182/blood-2014-10-607341 PMC4915792

[bib22] Bennett JM, Catovsky D, Daniel MT, Flandrin G, Galton DA, Gralnick H et al. The chronic myeloid leukemias: guidelines for distinguishing chronic granulocytic, atypical chronic myeloid, and chronic myelomonocytic leukaemia. Proposals by the French-American-British Cooperative leukaemia group. Br J Haematol 1994; 87: 746–754. 7986717 10.1111/j.1365-2141.1994.tb06734.x

[bib23] Cheson BD, Greenberg PL, Bennett JM, Lowenberg B, Wijermans PW, Nimer SD et al. Clinical application and proposal for modification of the International Working Group (IWG) response criteria in myelodysplasia. Blood 2006; 108: 419–425. 16609072 10.1182/blood-2005-10-4149

[bib24] Meldi K, Qin T, Buchi F, Droin N, Sotzen J, Micol J-P et al. Specific molecular signatures predict decitabine response in chronic myelomonocytic leukemia. J Clin Invest 2015; 125: 1857–1872. 25822018 10.1172/JCI78752 PMC4611703

[bib25] DePristo MA, Banks E, Poplin R, Garimella KV, Maguire JR, Hartl C et al. A framework for variation discovery and genotyping using next-generation DNA sequencing data. Nat Genet 2011; 43: 491–498. 21478889 10.1038/ng.806 PMC3083463

[bib26] Traina F, Visconte V, Elson P, Tabarroki A, Jankowska AM, Hasrouni E et al. Impact of molecular mutations on treatment response to DNMT inhibitors in myelodysplasia and related neoplasms. Leukemia 2014; 28: 78–87. 24045501 10.1038/leu.2013.269

[bib27] Bejar R, Lord A, Stevenson K, Bar-Natan M, Pérez-Lagada A, Zaneveld J et al. *TET2* mutations predict response to hypomethylating agents in myelodysplastic syndrome patients. Blood 2014; 124: 2705–2712. 25224413 10.1182/blood-2014-06-582809 PMC4208285

